# Experiences of accreditation impact in general practice – a qualitative study among general practitioners and their staff

**DOI:** 10.1186/s12875-019-1034-4

**Published:** 2019-10-28

**Authors:** Marius Brostrøm Kousgaard, Thorkil Thorsen, Tina Drud Due

**Affiliations:** The Research Unit for General Practice and Section of General Practice, Department of Public Health, University of Copenhagen, Øster Farimagsgade 5, 1014 Copenhagen, Denmark

**Keywords:** Accreditation, General practice, Impact, Quality standards, Qualitative study

## Abstract

**Background:**

Accreditation is a widespread tool for quality management in health care. However, there is lack of research on the impact of accreditation, particularly in general practice. This study explores how general practitioners and their staff experienced the impact of a mandatory accreditation program in Denmark.

**Methods:**

Qualitative interviews with general practitioners and staff from 11 clinics. The respondents were interviewed twice: during preparation and after the survey visit. The interviews were analyzed using thematic analysis, and all specific changes and other types of impact were extracted from the transcribed interview data from each clinic.

**Results:**

The impact of accreditation varied markedly among the clinics as did the participants’ overall assessments of accreditation. Concerning specific changes in behavior and physical infrastructure, some clinics had only implemented a few minor changes in response to accreditation, some had made a relatively moderate number of changes, and a few clinics had made relatively many changes including a few pronounced ones. Further, some participants experienced that accreditation had enhanced knowledge sharing or upgraded competencies, and increased job satisfaction. However, the workload related to accreditation was emphasized as a problem by a majority of the professionals and for a few, accreditation had influenced job satisfaction negatively.

**Conclusion:**

Accreditation may affect general practice clinics in very different ways. In spite of several examples of positive impact, the results suggest that it is difficult to design a mandatory accreditation program for general practice in which most professionals experience that the benefits of accreditation equal the resources used in the process.

## Background

Accreditation is a process in which an accreditation agency conducts a systematic assessment of an organization based on a set of quality standards. This usually involves a formal survey visit at the organisation after which the accreditation agency makes a decision on the granting of accreditation status to the organisation. Accreditation has been introduced in many health systems across the world as an instrument for quality control and quality improvement [1]. The basic idea is that the target organisations improve on quality and patient safety by seeking adherence to the requirements of the accreditation standards during the process of preparing for the survey visit and/or in response to the assessment of the surveyors.

Accreditation has a long history in the secondary sector, but has also been adopted in general practice in countries such as Australia, New Zealand and the Netherlands [2, 3]. Participation in general practice accreditation is usually voluntary for the clinics, but in some countries it is compulsory [2].

Reviews of the literature have pointed to several gaps in the knowledge base on accreditation despite of the widespread use and the substantial expenses associated with accreditation [4]. In the hospital sector there is a shortage of high-quality studies on the health effects of accreditation on patients [4–6]. In general practice, there is a pronounced lack of empirical research on the implementation and impact of accreditation [3] and the sparse evidence on impact is inconclusive. Two before-after studies from Germany and Switzerland reported improvements in practice management from participation in voluntary accreditation under the European Practice Assessment program [7, 8]. However, these studies had important methodological limitations and did not include clinical outcomes. A comparative observational study from Dutch general practice measured the effects of accreditation on chronic care but could only attribute few improvements to accreditation [9].

A few qualitative studies have reported some positive consequences of accreditation in general practice, particularly in the area of patient safety [10, 11]. However, these studies did not explore and describe impact at the clinic level in detail. At the same time, studies have found that professionals from general practice are concerned about the administrative burdens of accreditation [12, 13], concerns which have also been raised by health professionals in other health care settings [1]. Hence, when investigating the impact of accreditation, it is important to pay attention to positive as well as negative experiences.

In Denmark, a national program of accreditation for general practice was rolled out from 2016 to 2018. The program was formally presented with an emphasis on quality improvement, but was nevertheless controversial upon its establishment particularly due to its mandatory nature. Thus, a survey among Danish general practitioners (GPs) prior to implementation showed that almost half of the respondents had negative attitudes towards accreditation and that negative attitudes were associated with perceiving accreditation as an external control tool and having concerns about the expected time expenditure involved in accreditation [14].

This study is part of a larger research project on the implementation and impact of accreditation in general practice in Denmark. The project involves both quantitative and qualitative approaches [15, 16]. While quantitative methods can produce more generalizable results on the effects of new interventions, qualitative investigations can, among other things, contribute with more detailed knowledge on the nature of intervention impact from the perspective of the professionals.

In this paper, we present the results from a qualitative study of how professionals from 11 general practice clinics in Denmark experienced the impact of accreditation. The paper identifies the various types of impact related to accreditation in the clinics and articulates the professionals’ assessments of positive and negative consequences of accreditation.

## Methods

### Setting and intervention: general practice and accreditation in Denmark

The health care system in Denmark is mostly tax financed and citizens can receive care from public hospitals and general practice free of per service charge. The responsibility for procuring health services at the hospitals and in general practice rests with the five Danish regions.

The large majority of general practice clinics are privately owned and their services are publicly reimbursed and regulated in the collective agreement entered by the Organisation of General Practitioners and the Danish Regions [17]. The income of the clinics is generated as a combination of fee-for-service and capitation. While most GPs work in partnership clinics owned by two or more GPs, single-handed clinics are still common (constituting about half of the clinics).

Accreditation was mandatory in general practice from 2016 to 2018. The institution responsible for carrying out accreditation was the Danish Institute for Quality and Accreditation in Healthcare (IKAS).

The accreditation standards for general practice comprised 16 standards with 64 associated indicators. The topics of the standards are shown in Table 1. The standard set was constructed by IKAS and representatives from the Danish Regions, the Organization of General Practitioners in Denmark, the Danish College of General Practitioners, the Danish Association of Practicing Medical Specialists, and Danish Patients.

**Table 1:** The 16 accreditation standards

Name of standard	Focus areas
1. The professional quality	Use of diagnosis coding. Collection, analysis and use of clinical data for quality improvement.
2. Use of good clinical practice	Adherence to clinical guidelines particularly for diabetes and COPD. Special attention to vulnerable patients via a yearly plan for a selected group
3. Adverse events	Reporting, follow-up and process for learning in case of adverse events.
4. Patient evaluations	Completion of a patient evaluation and follow-up on the results.
5. Prevention of confusion of patient’s identity	Identification of patients principally by social security number and labelling of diagnostic material.
6. Prescription of medicine and renewal of prescriptions	Rational and safe medicine ordination and renewal of prescriptions. Participation in regional initiatives for correct medicine management. Annual assessment of patients’ list of medicine. Reporting of side effects.
7. Paraclinical tests (blood samples, urine samples, histological tests, smear tests, microbiological tests and diagnostic imaging tests)	Execution of tests and handling of test materials. Quality control of equipment. Requisition and follow-up of paraclinical tests. Procedures for test results in case of GP’s absence. Procedures for missing tests results.
8. Emergency response and cardiac arrest	Handling of acute disease and cardiac arrest in the clinic. Regular control of emergency equipment and medicine (functionality, accessibility and expiry dates). Documentation of participation in cardiopulmonary resuscitation course within the last three years.
9. The patient health record, data safety and confidentiality	Content of patient health record conforms to current legislation. Journal audit performed and followed-up upon if needed. Safe storage, handling and destruction of sensitive personal data. Discretion and confidentiality for patients.
10. Accessibility	Accessibility in accordance with the collective agreement (e.g. telephone hours, opening hours and waiting time). Physical accessibility. Visitation of patients. Online practice declaration with relevant information.
11. Referral	Relevant and adequate content and handling of referrals.
12. Coordination of patient care	Coordination and continuity of patient trajectories in the clinic and in collaboration with other health care providers.
13. Acquisition, storage and disposal of clinical utensils and medicine/vaccines	Sufficient stuck of utensils, medicine and vaccines. Correct storage of medicine e.g. at the right temperature. Control of expiry dates. Correct disposal.
14. Hygiene	Cleaning of the clinic and inventory. Cleaning and storage of medical equipment. Correct hand hygiene. Management of infectious patients.
15. Management and operations	Ensuring good management via plans for quality improvement, division of responsibilities and tasks, quality control and development goals.
16. Hiring, introduction and competency development	Procedures for employing new staff with the right competences, for introducing new doctors and staff, for supervising staff and doctors in training and for ensuring on-going competency development.

Each standard contained a description of its overall purpose, scope and requirements along with a number of indicators describing what areas and activities the clinic should be able to account for at the survey visit. The standards also contained references to guidelines and other documents to be used to explore the requirements in more detail. Although the standards were set within a regulative frame (mandatory accreditation) they were generally formulated in a relatively open way in order to stimulate team reflections on quality improvement in the clinics [18].

For their participation in the accreditation program the clinics received 20.000 Danish kroner (approx. EUR 2650) per GP in the practice. Half of this amount was settled at the beginning of the process and the rest when the practice was accredited.

Having received notification of the date of their survey visit, the clinics had 1 year to prepare for the visit. During this period of accreditation various agencies offered some kind of support to clinics preparing for the survey visit [16]. Approximately 1 year after the notification date, the clinics received a survey visit by two surveyors who questioned the GPs and the staff to determine whether the clinic adhered to the accreditation standards. One surveyor was a GP (active or retired), and the co-surveyor had a background in health care and often experience from general practice (e.g. nurse). The survey visit was scheduled to last about 4 hours in solo practices and extra hours could be added in clinics with more GPs. For practical reasons the clinics usually closed down during the visit.

After the survey, the clinics received the surveyors’ summarized report to which they could make objections in case of misunderstandings. Subsequently the accreditation agency decided on the granting of accreditation status (accredited, accredited with remarks, not accredited). In order to receive accreditation, some clinics had to go through a follow up process via phone or via an additional survey visit.

### Study participants

The study was conducted in two administrative regions (the Capital Region and the Region Zealand) and included qualitative interviews with GPs and staff from 11 general practice clinics (totaling 37 participants). Two different regions were chosen to achieve some geographical variation. The Capital Region is the most densely populated region with the large majority of the population located in city areas in or around Copenhagen. Region Zealand covers a more rural area, less densely populated with many provincial towns. The clinics were sampled strategically [19] with regards to variations in geography, practice type (solo/partnership) and GPs attitudes towards accreditation reported in a previous survey [14]. The sampling occurred among clinics that were scheduled for survey visits in 2017. The clinics were contacted via e-mail and telephone. Initially, we included 12 clinics in the study but one clinic had to be excluded, due to their survey date being postponed. Information on the clinics and respondents can be found in Table 2. Among the GPs in the study, 12 were males while 7 were females. The staff respondents were all female. In each clinic, all GPs and staff were sampled for inclusion in the study. In some clinics it was not possible for all GPs and staff to be present at the interviews, and in one clinic a staff member did not wish to participate due to the stress and negative feelings associated with accreditation.

**Table 2:** Clinics and respondents recruited for the study

Clinic	Type of clinic	GPs and staff in the clinic	Respondents at the first interview	Respondents at the second interview	A priori attitude to accreditation (GPs)
1	Partnership	3 GPs, 1 nurse, 2 secretaries	2 GPs, 1 nurse, 1 secretary	1 GP, 1 nurse, 1 secretary	Negative
2	Solo	1 GP, 2 nurses	1 GP, 2 nurses	1 GP, 2 nurses	Positive
3	Partnership	3 GPs, 2 nurses, 3 secretaries	3 GPs, 2 nurses, 1 secretary	3 GPs, 2 nurses, 1 secretary	Negative
4	Solo	1 GP, 1 biomedical laboratory scientist	1 GP, 1 biomedical laboratory scientist	1 GP, 1 biomedical laboratory scientist	Positive
5	Solo	1 GP, 1 secretary	1 GP, 1 secretary	1 GP, 1 secretary	N.A.
6	Partnership	3 GPs, 3 nurses, 1 secretary	3 GPs, 2 nurses, 1 secretary	3 GPs, 2 nurses, 1 secretary	Positive
7	Solo	1 GP, 1 nurse	1 GP	1 GP	Negative
8	Partnership	2 GPs, 2 nurses	2 GPs, 2 nurses	2 GPs, 2 nurses	Negative
9^a^	Partnership	2 GPs, 1 secretary	2 GPs	–	Positive
10	Solo	1 GP, 1 nurse	1 GP, 1 nurse	1 GP, 1 nurse	Negative
11	Solo	1 GP, 1 nurse	1 GP, 1 nurse	1 GP, 1 nurse	Positive
12	Partnership	3 GPs, 2 nurses, 2 secretaries	3 GPs, 2 nurses	3 GPs, 2 nurses	Negative^b^ Positive^b^

^a^The clinic was excluded from the study due to postponement of survey visit

^b^Two different GPs had answered the questionnaire

As it appears from Table 2, most of the practice staff in the clinics were nurses and secretaries. Nurses in general practice performs a variety of tasks such as chronic disease check-ups, gynecological examinations, wound treatment, lung function tests and blood sample tests. In the clinics that did not have a secretary, nurses also performed secretary work. In general practice, secretary work usually consists of answering queries from patients and making appointments, preparing prescription renewals, receiving patients in the clinic, taking blood samples, ordering equipment etc. The biomedical laboratory scientist in the study performed both nursing tasks and secretary tasks.

### Qualitative interviews

In order to identify potentially important themes for the interview guides, pilot-interviews were conducted with practitioners from two clinics that had already gone through the accreditation process.

Subsequently, professionals from the 11 clinics were interviewed twice: one interview while the clinics were in the process of preparing for the survey visit (3–8 months before the survey); and one interview 2–7 months after the survey visit. In total, 42 interviews were performed. The interviews lasted from 30 to 75 min with a meantime of 50 min. MBK and TD performed the interviews.

GPs and staff were interviewed separately in order to make room for conflicting views between employers and employees concerning the preparation process and the perceived value of accreditation in the clinic.

The first round of interviews (performed in the preparation phase) mainly focused on the challenges of preparing for accreditation as well as on identifying the accreditation related changes already made in the clinics. The second round of interviews (performed after the survey visit) mainly focused on how the participants perceived the impact of accreditation in terms of specific changes and overall consequences of accreditation (positive and negative). The interview guide also included questions regarding the participants’ experiences of the survey visit and any changes resulting from the visit. During the interviews we placed an overview of all the standards on the table to assist the respondents in recalling the individual standards and any changes made in relation to these.

The interview approach was semi-structured and the interview guides were adjusted a few times to include emerging perspectives and potentially important issues.

All participants were offered anonymity and informed that identifiable information would not be submitted to any third parties.

### Analysis

All interviews were audio recorded and transcribed. The interview material was analyzed using thematic analysis [20] and the software program NVivo. All authors took part in coding and analyzing the material. In order to achieve overview and immersion we first read and summarized each of the interviews. Then we decided on a coding structure which we applied on two interviews. After comparison and discussion, we adjusted the coding structure, coded the rest of the interviews, and drafted summaries for each clinic based on the coded material from both interview rounds. In this way we were able to delineate and compare the various types of changes made in the clinics, and relate the changes to the accreditation standards along with the respondents’ assessments of other consequences of accreditation in the clinic. The mandatory one-time activities required by some of the standards (performing a patient evaluation and a journal audit, participating in mandatory course in cardiopulmonary resuscitation and selecting a vulnerable group to focus on) were not coded as changes in themselves but any specific change in practice which these activities gave rise to were (e.g. changing the phone system in response to the patient satisfaction survey). If the respondents experienced that these one-time activities by themselves had affected their knowledge or competences this was coded as a type of impact but not as a specific change (see also Additional file 1). In the result section we report on both the specific changes and other perceived impact of accreditation.

## Results

The experiences of the impact of accreditation in the 11 clinics could be divided into four different areas of impact: 1) Behavior and physical infrastructure, i.e. the specific changes implemented in the clinics in response to accreditation; 2) Knowledge and competencies; 3) Resources; 4) Job satisfaction. After presenting the findings within these areas of impact, we outline the professionals’ overall assessments of accreditation.

### Impact on behavior and physical infrastructure

The table in Additional file 1 presents the specific changes implemented in each of the clinics as part of the accreditation process according to the respondents.

Most changes in the clinics were made during the preparation phase (and not in response to the surveyors’ assessments of the need for change). When preparing for the survey visit, each clinic decided which changes to implement in relation to the standards. The clinics interpreted the standards in different ways and their decisions were influenced by their overall approach to accreditation, i.e. a positive occasion for change vs. a bureaucratic task that had to be completed with as little effort as possible (or something in between) as well as by their different expectations about what would be sufficient to achieve accreditation. Therefore, the number and types of changes made in a particular clinic did not necessarily reflect a number and type of ‘objective gaps’ between the 16 accreditation standards and the state of affairs in the clinic before accreditation, and some clinics had made more changes than what was required to achieve accreditation status. Thus, the fact that a clinic had made relatively many changes did not necessarily mean that the clinic provided a low level of quality (in terms of the accreditation standards) prior to the preparation for accreditation.

The table in Additional file 1 shows that a large number of different changes had been implemented across the 11 clinics. One type of change concerned the physical infrastructure of the clinic, including re-design of clinic spaces (e.g. signs to signal areas of restricted access; discretion lines on the floor; creation of visibly marked ‘clean zones’ and ‘unclean zones’) and acquisition of new medical and administrative equipment/technology (e.g. an autoclave; a refrigerator; clothes for interacting with contagious patients; cabinets for safekeeping of medicine; extra disinfectant dispensers, a phone system with queue function). Another type of change concerned professional and administrative behaviors, such as adjusting procedures for sterilization of medical equipment; checking refrigerator temperature and expiry dates for medicine more systematically; asking more systematically for patients’ social security number; introducing new routines for ensuring data security.

Many changes were minor changes in the sense that they were relatively simple to implement, e.g. locking the computer screen when leaving the consultation room, removing patient identifiable information from public view, updating the emergency box. Other changes were more complex and/or time-consuming such as making new procedures for following up on paraclinical tests or planning and implementing a focused effort for a vulnerable patient group.

The data showed large variations between the 11 clinics concerning the number and types of changes implemented in response to accreditation (cf. Additional file 1). For example, Clinic 1 and Clinic 11 had only performed a few minor changes. Other clinics had performed a moderate number of changes of varying nature (e.g. Clinic 4, Clinic 10). Finally, two clinics (Clinic 6 and Clinic 12) had performed a larger number of changes, including some that were relatively pronounced in terms of resource use. In Clinic 6, a nurse had taken on a new outgoing role in relation to vulnerable elderly patients, and Clinic 12 had changed its prescription procedure so that patients always had to get an appointment in the clinic when renewing their medication. This clinic had also hired a new secretary to reduce phone waiting times (in response to the results of the patient satisfaction survey).

Certain work areas were much more affected by accreditation than others in terms of the specific changes implemented. Hence, as shown by Table 3, some standards were associated with changes in several clinics while other standards were associated with no or few changes in the clinics. For most of these latter standards, the professionals believed that the clinic was already in compliance with the requirements. Most clinics also deemed that they were in compliance with the requirement on the use of clinical guidelines for diabetes and COPD (Standard no. 2) as this had been a focus area for many years in general practice. Still, some adjustments did occur in relation to chronic care management: In a few clinics the standard had stimulated a dialogue that resulted in a few clarifications in the division of tasks between GPs and staff, and two GPs reported they had taken a more systematic approach to calling in patients for check-up consultations. Further, in some clinics the standard had inspired a more focused effort for a selected vulnerable patient group. Other clinics had mainly regarded and presented existing activities in the clinic as being in adherence with the requirement concerning vulnerable groups.

**Table 3:** Changes made in the clinics per standard

Name of standard	Changes made (with clinic identifiers in parenthesis)
1. The professional quality	• Increased and improved use of diagnosis coding in the patient records (6, 7).
2. Use of good clinical practice	• More systematic call-in of patients with COPD and diabetes for check-ups (2, 7). • Clarification of task division between GPs and nurses in diabetes and COPD procedures (4, 10, 12). • Purchased a new spirometer (2). • Preventive and follow up home visits by a nurse to elderly patients (6). • Increased focus on dementia patients (7, 8). • Clearer task division regarding patients with psychiatric problems and alcohol and drug abuse (12).
3. Adverse events	• Found out how to report adverse events and have reported some (4). • Adverse events have become a regular topic at monthly meetings (8).
4. Patient evaluations	• Changed telephone system to one with queue function (1, 10). • Improved entry for wheelchair users (1). • GP closes the office door in the morning when having phone consultations (2). • Water now available in the waiting room (4).
5. Prevention of confusion of patient’s identity	• Ask more often for the patient’s social security numbers when performing tests (2, 3, 5, 6, 7, 8, 10, 11, 12). • GP places test-samples and social security number together in small boxes and the nurse labels them (before they were placed on the table together, but with the risk of being mixed up) (10). • Always label a test sample container with the patient’s social security number before putting it aside (12).
6. Prescription of medicine and renewal of prescriptions	• A clearer division of tasks when prescribing medicine (6). • New procedure for renewal of medicine, where patients can only renew some types of medicine prescriptions by having a consultation in the clinic (12).
7. Paraclinical tests	• Use of reminder functionality in the computer system to ensure monitoring of incoming test results and patients getting the result (3, 4, 6, 8, 12). • Introduced a paper and pencil system to monitor received feedbacks of test results (2). • After a paraclinical examination patients with chronic diseases are now scheduled to a consultation with the GP (2). • Clearer task division in the clinic regarding reception and forwarding of test results (3). • New, clear procedures to ensure that deviant test result are delivered to the patient e.g. by keeping and regularly checking a copy of the test requisition (5, 8, 10, 11). • All test results (deviating as well as normal) are now given to the patients (previously only deviating results) (7, 8). • Introduced a procedure for checking that test samples from the clinic have reached the laboratory (6). • Stopped cultivating urine samples themselves, now sending the samples to the lab instead (12).
8. Emergency response and cardiac arrest	• Made or updated emergency medicine box (3,4, 5, 6, 7, 12) • Procured information on correct intervals for control and renewal of the defibrillator and made a schedule with fixed timespans for future controls (10). • Purchased a heart defibrillator (12).
9. The patient health record, data safety and confidentiality	• No papers with social security numbers or patient records are visible lying around (3, 5, 6, 7, 12). • Stopped mentioning social security number on the phone or at the secretary’s desk (2, 6). • No name or social security numbers on things thrown in the garbage (5). • Lock the computer screen when exiting a room (1, 4, 6). • Lock cabinets in the patient waiting area (8). • Signs on the doors marking no entrance allowed (1, 6). • GP closes the office door in the morning when having phone consultations (2). • Placed a discretion line on the floor in front of the secretary’s desk (3). • More frequent use of shredder (5, 12). • Added a code to insert in the patient record indicating informed consent (4). • More meticulous registration of CAVE in the patient record (4, 6). • Copy medicine list from the electronic medicine module to the patient record to comply with record keeping obligation (6).
10. Accessibility	• Changed telephone system to one with queue function (1, 10). • Engaged an extra part time secretary to ease patients’ phone access (12). • Opened a Facebook site with news and information (12). • Improved entry for wheelchair users (1).
11. Referral	• Update the electronic medicine module more frequently when referring patients to hospitals (6).
12. Coordination of patient care	
13. Acquisition, storage and disposal of clinical utensils and medicine/vaccines	• More systematic control of expiration dates of medicine and vaccines, and of refrigerator temperature (3, 5, 6, 7, 10, 11, 12). • Purchased an electronic thermometer with data-logging for measuring refrigerator temperature giving continuous temperature overview and alarms in case of critical fluctuations (4, 6). • Replaced old refrigerators with a new one (6). • Cleaned up medicine room and medical bag and disposed of the expired medicine (5, 6). • Regular control of medical bag (10). • Store medical bag so it is inaccessible to patients (4). • More correct disposal of utensils and vaccines (6, 12). • Thorough clean-up of equipment (e.g. disposal of syringes past expiration date) (7, 10).
14. Hygiene	• Toys completely removed from the waiting room or thrown out toys not suitable for the dishwasher, while cleaning remaining toys weekly (1, 12). • Replaced a normal oven with an autoclave for sterilization (3, 5). • More systematic and frequent controls of the autoclave (4, 6). • Added disinfection between washing of instruments and autoclavation (2, 4, 10). • Use bags for storage of instruments after sterilization (4, 8). • Purchased a new dishwasher for cleaning instruments (8). • More spirit dispensers and/or use more spirit (3, 6, 7). • Use a log sheet when performing systematic controls e.g. of tape for sterile utensils (1). • More fixed time intervals for control of equipment (3). • Changed open cabinets for storing utensils with closed ones (3). • Blood pressure monitors lent to patients for measurement at home are now cleaned every time they are returned to the clinic after use (6, 12). • Use more single-use equipment or changed to only single-use equipment (6, 12). • More frequent cleaning of chairs and examination couch (4, 5, 12). • Daily cleaning and control of the toilets (4). • Well defined areas and clearer labeling of clean and unclean surfaces (6, 8) • Purchased clinic clothes with short sleeves (used their own clothes before) (6). • Do not use wristwatches (6). • Have two types of gloves (6). • Acquired special suits to use in case of contagious patients (5, 10). • Set up a room for handling contagious patients and a procedure for scheduling them at the end of the day (12). • Installation of a hand dryer instead of towels at the patients’ toilet (12). • Thorough cleaning of the clinic before the survey (5, 11).
15. Management and operational activities	• Made an annual planning wheel displaying tasks in the clinic on a monthly basis (6, 8, 12).
16. Hiring, introduction and competency development	• Made an introduction program for new staff (12).

As seen in Table 3, the standards associated with specific changes in most clinics concerned issues of data security and discretion (standard no. 9), storage and control of medicine and vaccines (standard no. 13), hygiene (standard no. 14), secure identification of patients (standard no. 6), procedures for following up on paraclinical test results (standard no. 7), and emergency response (standard no. 8). Several respondents experienced that going through the accreditation process had instilled more systematics into one or more of these areas, which the standards had called attention to:

*“We have become more structured. If you look at the board [the annual wheel showing a set of tasks to be performed each month and the person responsible], you see a lot of things that we already did, but not with the same frequency. Now, we are more structured, so that we don’t miss anything: we follow up on the smears tests, we follow up on the vaccinations …* “*[Staff, Clinic 6].*

Some of the changes made – like those related to more secure storage of medicine, increased cleaning and disinfection, or documenting the monitoring of refrigerator temperature and medicine expiry dates – were usually not seen as having much importance for the patients or the clinic since the respondents did not believe that particular problems existed in these areas:

*“We disinfect more now but [our processes] were not dangerous before [ …] we have never had any infections” [Staff, Clinic 2].*

However, changes in other areas were considered to benefit patients to varying degrees, e.g. changes in discretion, accessibility, attention to vulnerable groups, and particularly, changes in some activities related to patient safety (paraclinical tests and prescriptions):

*“[due to] the change we have made, so that the patients have to come in to receive their medication [ …*], *some patients have discontinued medication because they did not need it anymore” [Staff, Clinic 12].*

In another clinic (no. 6), the GPs related that they had become more attentive to ensure a match between the patient in the room, the ‘patient on the screen’, and ‘the patient on the requisition note’ when taking specimen for analysis, and they believed that this would reduce the risk of adverse events.

### Impact on knowledge sharing and competencies

According to several respondents, working with the accreditation standards had some positive impact that was less tangible than the specific changes in behavior and physical infrastructure.

First, in some clinics the discussions and reflections of current practice prompted by the standards had provided the professionals with increased knowledge of each other’s work. For some respondents such insight was mostly a matter of ‘nice-to-know’, but particularly for some staff, it was seen as having concrete advantages. Here a staff member explains how having more knowledge about the GPs’ work was helpful when answering patient queries on the phone:

*“It means a lot for me when I am sitting on the phone all morning [ …*] *to have an idea of what they [the patients] have been told [by the GP]. So I can say ‘well, what the GP meant was so and so’. I can be more self-assured when I talk to people because I know [what they have been through with the doctor].” [Staff, Clinic 4].*

Second, some respondents believed that the accreditation process had improved their capabilities (individually and as a team) for engaging in quality improvement activities in the future by making them more aware about where to look for relevant information, more aware of how to structure discussions about quality in the clinic, or better at comparing their own practice with official quality standards.

Third, some respondents stated that working with the standard on emergency response (Standard no. 8) had served to brush up their individual competencies in this area (the standard required that all GPs and staff in the clinic had attended a course in cardiopulmonary resuscitation within the last 3 years). This was experienced as reassuring and potentially important although acute situations were rare.

Finally, several respondents mentioned that the documents describing work flows and task division (drafted during the accreditation process) could be seen as a new knowledge resource which could be used to facilitate the introduction of new personnel in the clinic. However, no specific experiences with this were reported.

### Impact on resources

The impact of accreditation on the clinics’ resources in terms of time expenditure was a central issue in the interviews. In the majority of the clinics, the respondents found that the high amount of time spent on the accreditation process was, to varying degrees, problematic. In two clinics (no. 3, 8), the GPs stated that their waiting lists had increased due to the time taken up by accreditation, and several respondents suggested that the time spent on accreditation could have been used for other quality improvement activities or for seeing patients:

*“Normally we use our weekly meeting for educational activities but that has been set aside during the last year. We have been tied up by having to go through all these things [the accreditation standards], instead of dealing with what we think is most relevant in the clinic. So, it has been problematic. And I have used too much of my spare time at home.*” [GP, Clinic 8].

Apart from the survey visit itself (for which the clinics had to allocate at least 4 hours during daytime), the time expenditure was related to the following elements of the accreditation process:

- Reading, understanding and discussing the standards and their implications for the clinic and describing practice procedures in formal documents. This work could include participation in regional information meetings and workshops (see also [16]);

- Performing the mandatory one-time activities required by the standards (no. 4, 8, 9): a patient evaluation; a journal audit; and attending a course in cardiopulmonary resuscitation.

- Implementing and sustaining the changes that each clinic decided were necessary to adhere to the standards (cf. above).

It was difficult for the respondents to estimate the exact amount of time spent on accreditation, but most GPs considered that the economic compensation did not cover their total expenses which in some cases also included expenses for new equipment. However, while the issue of time was important, the monetary aspect in itself was not a key concern among the GPs.

### Impact on job satisfaction

The impact of accreditation on the professionals’ job satisfaction varied. In three clinics the respondents reported that accreditation had influenced their job satisfaction negatively due to the extra work involved in the process (cf. above), and in a few cases also because the respondents experienced that the prospect of having their work scrutinized at the survey visit was stressful in itself. The respondents in these clinics had generally been negative towards accreditation from the outset and regarded accreditation as an external control tool. In one clinic, a nurse had to take absence from work for a month shortly after the survey visit due to stress related symptoms. In another clinic, the GP was generally dissatisfied with the working conditions in general practice due to increased time pressure and public regulation, and the introduction of mandatory accreditation was the last straw that had made her decide to retire earlier than planned:

*“Our working day is being filled with routines of questionable relevance … all the things you have to document and document and document …*” *[GP, Clinic 7].*

Contrary, other professionals – particularly among the staff – experienced that accreditation had influenced their job satisfaction positively for one or more of the following reasons: Because the process of preparing had been a good collaborative experience involving increased ongoing dialogue between GPs and staff about the procedures in the clinic; and/or because some of the specific changes (e.g. in relation to hygiene and clearer divisions of work) were seen as improvements that would not have been made without accreditation; and/or because working with the standards had confirmed that they were doing the right things in the clinic and/or because the survey visit and the final accreditation approval had served as an external validation that the clinic was performing well:

*I think that I have become more content … It is pleasing to know that you are working in a place where we are in control of things [Staff, Clinic 8].*

### Overall assessments

There were large differences between the respondents as to how they assessed the value of accreditation in relation to the time expenditure involved in the process.

Generally, respondents from clinics in which one or more GPs had been positive about accreditation from the outset, were more positive in their assessments of the accreditation process and its impact than respondents from clinics where the GPs had been skeptical about accreditation from the outset.

In four clinics (no. 4, 5, 11, 12), the respondents generally assessed that going through the accreditation process had been worth the effort. While one of these clinics (no. 11) had only made few improvements, respondents in the three other clinics suggested that accreditation had resulted in several improvements, e.g. in relation to patient safety. For example, a GP reflected that working with the standard on emergency response (by attending the mandatory courses, formulating new instructions, and re-arranging the medicine in the fridge) had proven useful in specific situations:

*“[a few times] we have had somebody with chest pains and then it was really nice to be able to go to the fridge knowing exactly where the medication was and having clear instructions so each of us knew what to do [ …*] *I have been pleased about that. I don’t think we would have done it [organized the emergency response as systematically] if we had not been forced to do it.” [GP, Clinic 4].*

In four other clinics, the overall assessments of the respondents were mixed: In Clinic 8, the GPs and the staff held contrasting views about the accreditation process and its significance in the clinic: The GPs, who had been negative about accreditation from the outset, considered the resource expenditure to be excessive compared to the few benefits. The staff, however, had not found the process burdensome and experienced more benefits than the GPs, particularly due to the hygienic related changes implemented in the clinic – changes which the staff had previously not been able to win acceptance for in the clinic. In Clinic 6, the professionals had been positive about accreditation from the outset and devoted much time and energy to the process. They had also implemented relatively many changes including some that were not strictly necessary to receive accreditation. Still, at the end of the process some of the respondents were unsure whether the benefits of the process were proportional to the effort they had invested. In Clinic 2 where the GP had been positive about accreditation beforehand, the respondents had become frustrated with accreditation particularly at the beginning of the process due to the time they had used on meetings and paper work away from the patients. However, the collaborative process had been a good experience where they had questioned their own procedures and made some adjustments, but they did not regard these adjustments as being particularly important except from those related to following up on paraclinical test results. In Clinic 10, where the GP had initially been negative about accreditation, both the GP and the nurse were ambivalent about accreditation at the end of the process: On the one hand they found the time expenditure to be problematic; on the other hand they reckoned that the benefits (in terms of increased systematics in activities and more structured professional discussions) would not have been realized without going through accreditation.

In the last three clinics (no. 1, 3, 7), where the GPs had expressed negative a priori attitudes towards accreditation, the respondents assessed that the time expenditure associated with the accreditation process could not be justified when compared to the results. In these clinics, the respondents considered the benefits of accreditation to be minimal:

*“There is no proportionality between the time we have used on this system and the effects, at least not seen from our view. It has been grotesque [ …*], *we are already really busy as doctors … and if you have to spent time on something like this, it has to be more focused … around the things that make sense in general practice” [GP, Clinic 3].*

At the end of this quote, the GP raises the issue of the relevance of the accreditation standards for general practice. Although the respondents generally believed that most of the standards were relevant and legitimate as such, several respondents also found some of the requirements of particular standards to be somewhat irrelevant and impractical (e.g. aspects of the hygiene and management standards; patient identification by social security number at each consultation; conducting a mandatory patient survey). Some GPs also pointed out that the standards did not address the most important aspects of general practice, namely the personal interactions with – and treatment of – patients, and some commented that it was very difficult to construct valid and operational standards for formally evaluating these aspects of quality in general practice.

## Discussion

In this qualitative study in 11 general practice clinics, we found substantial variations in the impact of accreditation as reported by the professionals. Some clinics had only implemented a few minor changes in response to accreditation, some had made a relatively moderate number of changes, and a few clinics had made relatively many changes including a few pronounced ones. Most of the specific changes in the clinics concerned adjustments in areas concerning: procedures for following up on paraclinical test results, secure identification of patients, emergency response, hygiene, storage and control of medicine and vaccines, data security, and discretion. In addition to the specific changes, some respondents experienced that accreditation had enhanced knowledge sharing, upgraded competencies in relation to emergency response and future quality improvement, and increased job satisfaction. However, the extra work related to accreditation was emphasized as a problem by a majority of the respondents, and in a few cases the prospect of an external control visit was a stress-factor that had impacted job satisfaction negatively. Overall, the respondents were divided in their assessments of accreditation; for some accreditation had been a positive process of fine-tuning the clinic in certain areas, for others accreditation had mainly been a fruitless experience which had used up scarce resources.

In line with the findings from the present study, a few other qualitative studies have suggested that accreditation in general practice can lead to improvements in patient safety activities [10, 11]. Studies have also reported that accreditation may strengthen teamwork in the clinic [13] and facilitate individual and collective learning since the processes of formally describing local procedures serve to articulate the tacit knowledge of clinic members [11]. Such ‘softer’ forms of positive impact were also mentioned by some participants in the present study, although it was not a predominant theme in our data.

The results on the negative assessments of accreditation partly echo a recent study from Dutch general practice, in which professionals were dissatisfied with the time costs of accreditation and the lack of direct impact on patient-caregiver interactions [13]. Also, in a pilot evaluation of a voluntary accreditation program in general practice in England, many participants had positive experiences with the program, but nevertheless recommended that the program should be improved by focusing more on creating direct value for the patients and less on having the clinics document their procedures and plans [11].

In terms of the workload imposed by accreditation, a previous study (among the same clinics as included in the present study) showed that several clinics had spent much time and energy on understanding the requirements of the standards and describing local work processes in writing [16]. In several of the change areas mentioned by the clinics in our study, increased specificity would seem feasible to apply in order to achieve a better balance between the benefits and the work load of accreditation as perceived by the clinics.

Although accreditation appears as a tool which can be used to promote certain changes in patient safety activities, the costs of accreditation schemes (at the administrative and the clinic level) also raise the question of whether the improvements associated with accreditation may be achieved by other means. For example, instead of disseminating accreditation standards for the clinics to make sense of in advance of a survey visit, the sense making work of the clinics may be reduced by systematic use of specialized personnel who visit the clinics to provide targeted guidance on specific issues (e.g. hygiene, data security, medication, chronic care, organization) based on existing guidelines and regulations. Some clinics had good experiences with visits from specialized consultants when preparing for accreditation [16], and in Denmark arrangements with medical consultants have existed for some time.

A more bottom-up based approach to quality improvement in general practice is the concept of ‘quality clusters’. Here the central idea is that GPs from different clinics join together in groups (clusters) where they define their own needs, goals and methods of quality improvement. Quality clusters have been implemented in Scotland and Wales [21, 22], and have recently been introduced in Danish general practice to replace accreditation [23].

### Strengths and limitations

To our knowledge, this study is so far the most detailed qualitative study of the impact of an accreditation program on general practice clinics. The study included two rounds of interviews with both GPs and staff from different types of clinics. The sample procedure also ensured that the study included professionals who were initially positive about accreditation as well as professionals who were initially negative. However, some limitations apply to the study. It is possible that some respondents might (more or less unwittingly) have over-reported or under-reported the impact of accreditation due to various psychological mechanisms. For example, respondents who initially held strong negative or positive attitudes towards accreditation might have, respectively, downplayed or overstated the benefits of accreditation due to ‘confirmation bias’ [24]. Further, respondents who spend a substantial amount of time on implementing changes in response to the accreditation standards might be inclined to over-report the benefits due to ‘effort justification bias’ [25]. By interviewing different individuals in the clinics (participant triangulation) at two different points in time and by asking about specific changes related to the various standards, we tried to mitigate such potential biases of individual respondents. It would have strengthened the study further if we could have compared the statements from the interviews with other kinds of data, e.g. register data, or data from observations of work routines in the clinic before, during, and after accreditation. And while we did bring a copy of the standards to the table to aid the memory of respondents, the time limits of each interview sometimes made it difficult to go through all of the standards one-by-one with the respondents. Therefore, some (minor) changes might not have been reported. Finally, the 11 clinics included in the study constitute a small sample compared to the total number of general practices in Denmark. So, although the study uncovered a substantial variety of experiences and we assessed that a satisfactory level of data saturation was achieved, it is possible that other experiences of accreditation impact could be found in the wider population.

## Conclusion

This study showed that accreditation may affect general practice clinics in very different ways both in terms of the impact on behavior and organizational infrastructure (number and types of specific changes implemented) and in terms of impact on knowledge sharing, competencies, and resources. Further, there were large variations in the professionals’ assessments of accreditation. Some professionals experienced that accreditation had been a driver for positive change while others found that the accreditation process had been too burdensome and of little relevance to improving clinical practice. The results reflect the large heterogeneity of general practice which entails that the same intervention may be received very differently from clinic to clinic with very different consequences. Although several kinds of positive impact were reported, the results also suggest that it is difficult to create a mandatory accreditation program for general practice in which most of the professionals find that the benefits of accreditation match the resources used in the process. This should lead to considerations about how to obtain a better balance between achieved improvements and work load in general practice accreditation, and about whether the improvements associated with accreditation can be obtained by other, less resource-demanding means. Still, the limitations of the study make it difficult to draw firm conclusions about the impact and value of general practice accreditation. Further research employing qualitative as well as quantitative methods is required to strengthen the evidence base on the costs and benefits of accreditation in general practice.

## Supplementary information

**Additional file 1.** Changes in behavior and physical infrastructure in the eleven clinics. This includes a table which presents an overview of the changes in behavior and physical infrastructure implemented in the participating clinics.

## Data Availability

The anonymized transcribed interviews from the current study are available from the corresponding author upon reasonable request.

## Abbreviations

COPD: Chronic Obstructive Pulmonary Disease
GP: General practitioner
IKAS: Danish Institute for Quality and Accreditation in Healthcare
KIF: The Foundation for Quality Informatics

## References

[1] Greenfield D, Braithwaite J (2008). Health sector accreditation research: a systematic review. Int J Qual Health Care.

[2] Lester HE, Eriksson T, Dijkstra R, Martinson K, Tomasik T, Sparrow N (2012). Practice accreditation: the European perspective. Br J Gen Pract.

[3] O’Beirne M, Zwicker K, Sterling PD, Lait J, Lee Robertson H, Oelke ND (2013). The status of accreditation in primary care. Qual Prim Care.

[4] Hinchcliff R, Greenfield D, Moldovan M, Westbrook JI, Pawsey M, Mumford V, Braithwaite J (2012). Narrative synthesis of health service accreditation literature. BMJ Qual Saf.

[5] Brubakk K, Vist GE, Bukholm G, Barach P, Tjomsland O (2015). A systematic review of hospital accreditation: the challenges of measuring complex intervention effects. BMC Health Serv Res.

[6] Flodgren G, Pomey MP, Taber SA, Eccles MP (2011). Effectiveness of external inspection of compliance with standards in improving healthcare organisation behaviour, healthcare professional behaviour or patient outcomes. Cochrane Database Syst Rev.

[7] Szecsenyi J, Campbell S, Broge B, Laux G, Willms S, Wensing M, Goetz K (2011). Effectiveness of a quality-improvement program in improving management of primary care practices. CMAJ.

[8] Goetz K, Hess S, Jossen M, Huber F, Rosemann T, Brodowski M, Künzi B, Szecsenyi J (2015). Does a quality management system improve quality in primary care practices in Switzerland? A longitudinal study. BMJ Open.

[9] van Doorn - Klomberg AL, Braspenning JCC, Wolters RJ, Bouma M, Wensing M (2014). Effect of accreditation on the quality of chronic disease management: a comparative observational study. BMC Family Pract.

[10] Debono D, Greenfield D, Testa L, Mumford V, Hogden A, Pawsey M, Westbrook J, Braithwaite J (2017). Understanding stakeholders’ perspectives and experiences of general practice accreditation. Health Policy.

[11] Campbell Stephen M, Chauhan Umesh, Lester Helen (2010). Primary Medical Care Provider Accreditation (PMCPA): pilot evaluation. British Journal of General Practice.

[12] Rakic S, Novakovic B, Stevic S, Niskanovic J (2018). Introduction of safety and quality standards for private health care providers: a case-study from the republic of Srpska, Bosnia and Herzegovina. Int J Equity Health.

[13] Nouwens E, van Lieshout J, Wensing M (2015). Determinants of impact of a practice accreditation program in primary care: a qualitative study. BMC Fam Pract.

[14] Waldorff FB, Nicolaisdottir DR, Kousgaard MB, Reventlow S, Sondergaard J, Thorsen T, Andersen MK, Pedersen LB, Bisgaard L, Hutters CL (2016). Almost half of the Danish general practitioners have negative a priori attitudes towards a mandatory accreditation programme. Dan Med J.

[15] Andersen MK, Pedersen LB, Siersma V, Bro F, Reventlow S, Søndergaard J, Kousgaard MB, Waldorff FB (2017). Accreditation in general practice in Denmark: study protocol for a cluster-randomized controlled trial. Trials.

[16] Due TD, Thorsen T, Kousgaard MB (2019). Understanding accreditation standards in general practice – a qualitative study. BMC Fam Pract.

[17] Pedersen KM, Andersen JS, Sondergaard J (2012). General practice and primary health care in Denmark. J Am Board Fam Med.

[18] Accreditation standards. https://www.ikas.dk/den-danske-kvalitetsmodel/ddkm-in-english/accreditation-standards/. Accessed 9 May 2019.

[19] Patton M (2002). Qualitative research & evaluation methods.

[20] Braun V, Clarke V (2006). Using thematic analysis in psychology. Qual Res Psychol.

[21] Smith GI, Mercer SW, Gillies JCM, McDevitt A (2017). Improving together: a new quality framework for GP clusters in Scotland. Br J Gen Pract.

[22] National Assembly for Wales (2017). Inquiry into Primary Care: Clusters.

[23] Klynger skal booste kvaliteten i almen praksis [Clusters are to boost the quality in general practice] (2018). Ugeskr Laeger.

[24] Nickerson RS (1998). Confirmation Bias: a ubiquitous phenomenon in many guises. Rev Gen Psych.

[25] Hill LG, Betz DL (2005). Revisiting the retrospective pretest. Am J Eval.

