# Supplementary material for: Experiences of accreditation impact in general practice – a qualitative study among general practitioners and their staff
Source: BMC Fam Pract. 2019 Oct 28;20:146. doi: 10.1186/s12875-019-1034-4 (PMC6819337; doi:10.1186/s12875-019-1034-4)
Supplement: Supplementary file 1 — Additional file 1. Changes in behavior and physical infrastructure in the eleven clinics. This includes a table which presents an overview of the changes in behavior and physical infrastructure implemented in the participating clinics. [file 12875_2019_1034_MOESM1_ESM.docx]

**Additional file 1: Changes in behavior and physical infrastructure in the eleven clinics**

The table below presents the changes in behavior and physical infrastructure in each clinic resulting from the accreditation process according to the participants. At the interviews, the participants were provided with a list of all the standards and asked the question: “If we look at the individual standards, can you tell me if you have made any changes in the clinic as part of the work with the standards?” Further questions were then posed to validate and elaborate the answers. The table only presents specific changes in behavior and infrastructure, and not cases where existing procedures were put into writing but otherwise unchanged, nor the impact of the standards on other aspects of the clinic such as knowledge and competencies, resources, job satisfaction (see the rest of the result section in the paper). Standards for which no changes are presented either imply that the clinic was already adhering to the standard, that the clinic got through the accreditation process without complete adherence to the standard, or that the respondents did not recall any changes made in response to this standard.

The number and name of each standard refers to the description of the standards in Table 1 in the main text.

| **Clinic 1** | |
| --- | --- |
| 4. Patient evaluations | Changed telephone system to one with queue function (due to long waiting times and difficulties getting through).  Improved entry for wheelchair users. |
| 9. The patient health record, data safety and confidentiality | Lock the computer screen when exiting a room.  Signs on the doors marking no entrance allowed. |
| 10. Availability | Changed telephone system to one with queue function (due to long waiting time and difficulties getting through).  Improved entry for wheelchair users. |
| 14. Hygiene | Clean toys in the dishwasher once a week (not systematically before) and thrown out toys not suitable for the dishwasher.  Use a log sheet when performing systematic controls e.g. of tape for sterile utensils. |
| **Clinic 2** | |
| 2. Use of good clinical practice (Diabetes, COPD and a specific vulnerable group) | More structure in calling patients in for chronic care check-ups.  Purchased a new spirometer. |
| 4. Patient evaluations | GP closes the office door in the morning when having phone consultations, because patient replied that they could hear the conversations from the waiting area. |
| 5. Prevention of confusion of patient’s identity | Ask more often for the patient’s social security numbers when performing tests. |
| 7. Paraclinical tests | After a paraclinical examination patients with chronic diseases are now scheduled to consult their GP (not only the practice nurse, and not only by telephone).  Introduced a paper and pencil system to monitor received feedbacks of test results. |
| 9. The patient health record, data safety and confidentiality | Stopped mentioning social security number on the phone.  GP closes the office door in the morning when having phone consultations so patients in the waiting area cannot hear what is being said. |
| 14. Hygiene | Added disinfection between washing of instruments and autoclavation. |
| **Clinic 3** | |
| 5. Prevention of confusion of patient’s identity | Nurses check patient’s social security numbers more often when performing tests, but not always in case of well-known patients. |
| 7. Paraclinical tests | Use of reminder functionality in the computer system to ensure monitoring of incoming test results and patients getting the result.  Clearer task division in the clinic regarding reception and forwarding of test results. |
| 8. Emergency response and cardiac arrest | Updated emergency medicine box. |
| 9. The patient health record, data safety and confidentiality | Placed a discretion line on the floor in front of the secretary’s desk.  Turn all papers with social security numbers upside down. |
| 13. Acquisition, storage and disposal of clinical utensils and medicine/vaccines | More systematic control of expiration dates of medicine and vaccines, and of refrigerator temperature. Previously not scheduled, but conducted when there was time. |
| 14. Hygiene | Replaced a normal oven with an autoclave for sterilization.  Changed open cabinets for storing utensils with closed ones.  More spirit dispensers and use a bit more spirit.  Set more fixed time intervals for control of equipment. |
| **Clinic 4**  This clinic was established in 2015 and thus relatively new at the time of the study. The first interview was conducted in 2016 and the second in the middle of 2017, a few months after the survey visit. The participants stated that they had the accreditation standards in mind when establishing and organizing the clinic. Hence, the reported changes should be seen within this frame. | |
| 2. Use of good clinical practice (Diabetes, COPD and a specific vulnerable group) | According to the GP no changes were made regarding patients with diabetes or COPD. According to the staff, some adjustments were made: a clearer task division between her and the GP, and use of a template of standard phrases for systematization of the chronic disease check-ups. |
| 3. Adverse events | Found out how to report adverse events and have reported some concerning blood test collection from the practice to the hospitals. |
| 4. Patient evaluations | Water now available in the waiting room. |
| 7. Paraclinical tests | Use reminder functionality in the computer system to ensure monitoring of incoming test results and to ensure patients getting the result. |
| 8. Emergency response and cardiac arrest | Placed relevant medicine in bags in the refrigerator. |
| 9. The patient health record, data safety and confidentiality | Always fill out for CAVE in the patient records.  Lock the computer screen when exiting the room.  Added a code to insert in the patient record after each consultation indicating informed consent. |
| 13. Acquisition, storage and disposal of clinical utensils and medicine/vaccines | Purchased an electronic thermometer with data-logging for measuring refrigerator temperature giving continuous temperature overview and alarms in case of critical fluctuations.  Store medical bag so it is inaccessible to patients. |
| 14. Hygiene | Added disinfection between washing of instruments and autoclavation.  Systematic control of the autoclave.  Use bags for storage of instruments after sterilization.  Daily cleaning of e.g. desks, chairs and examination couch.  Daily cleaning and control of the toilets. |
| **Clinic 5** | |
| 5. Prevention of confusion of patient’s identity | Secretary asks the patient for social security number when performing a test (instead of going to the other room and check it in the patient record). |
| 7. Paraclinical tests | Keep a copy of the test requisition form and checking on a regular basis that patients with deviant test results have been informed. |
| 9. The patient health record, data safety and confidentiality | No papers with social security numbers or patient records lying around.  No name or social security numbers on things thrown in the garbage.  More frequent use of shredder. |
| 13. Acquisition, storage and disposal of clinical utensils and medicine/vaccines | More systematic control of expiration dates of medicine and vaccines, and of refrigerator temperature plus documentation in log lists.  Checked the medicine cabinet and medical bag for expired medicine. |
| 14. Hygiene | Acquired special suits to use in case of contagious patients.  Replaced a normal oven with an autoclave for sterilization.  More frequent cleaning of chairs and examination couch.  Thorough cleaning of the clinic before the survey. |
| **Clinic 6** | |
| 1. The professional quality | Nurses more aware of using diagnosis coding in the patient records more correctly. |
| 2. Use of good clinical practice (Diabetes, COPD and a specific vulnerable group) | Preventive and follow up home visits by a nurse to elderly patients (their chosen vulnerable group). |
| 5. Prevention of confusion of patient’s identity | Nurses always ask for patient’s social security numbers when performing tests. GPs ask for social security numbers more often when performing tests. |
| 6. Prescription of medicine and renewal of prescriptions | A clearer division of tasks when prescribing medicine. |
| 7. Paraclinical tests | Introduced a procedure for checking that test samples from the clinic have reached the laboratory.  Use of reminder functionality in the computer system to ensure monitoring of incoming test results and patients getting the result. |
| 8. Emergency response and cardiac arrest | Improved the emergency medicine box by sorting medicine in bags according to type of emergency and attached descriptions of what medicine to give the patient. |
| 9. The patient health record, data safety and confidentiality | More meticulous registration of CAVE in the patient record.  Copy medicine list from the electronic medicine module to the patient record to comply with record keeping obligation.  Lock the computer screen when exiting the room.  No papers with social security numbers lying around.  Box with letters to and from patients moved behind the secretary’s counter.  Signs on doors marking where entrance is not allowed.  The secretary does not ask for social security number in the reception, but gives the patient a piece of paper to write it on. |
| 11. Referral | Update the electronic medicine module more frequently when referring patients to hospitals. |
| 13. Acquisition, storage and disposal of clinical utensils and medicine/vaccines | More systematic control and documentation of expiration dates of medicine and vaccines.  Notation of date of first use of medicine.  Cleaned up medicine room and disposed of the expired medicine.  More correct disposal of utensils and vaccines.  Replaced old refrigerators (some with unstable temperature and frost on the back) with a new one.  Purchased an online thermometer with alarm function for the refrigerator.  Updated the emergency medicine box. |
| 14. Hygiene | Purchased clinic clothes with short sleeves (used their own clothes before).  Do not use wristwatches.  Have two types of gloves.  More spirit dispensers.  More systematic and frequent controls of the autoclave.  Blood pressure monitors lent to patients for measurement at home are now cleaned every time they are returned to the clinic after use.  Use more single-use equipment.  Clearer labeling of clean and unclean surfaces. |
| 15. Management and operational activities | Made an annual planning wheel depicting monthly tasks, each person’s areas of responsibility and log lists for ticking off completed tasks. |
| **Clinic 7** | |
| 1. The professional quality | Increased use of diagnosis coding in the patient records. |
| 2. Use of good clinical practice (Diabetes, COPD and a specific vulnerable group) | More systematic call-in of patients with COPD and diabetes for check-ups.  Special focus on patients where the GP suspects dementia. With the patient’s permission, the GP traces contact persons, home care, and refers to further examination. |
| 5. Prevention of confusion of patient’s identity | Ensure that the right label with patient identification is printed when performing tests (most often by asking the patients to confirm the social security number, otherwise by facial recognition). |
| 7. Paraclinical tests | All test results (deviating as well as normal) are now given to the patients (previously only deviating results). |
| 8. Emergency response and cardiac arrest | Made boxes with medicine for emergency situations. |
| 9. The patient health record, data safety and confidentiality | Ensure that no papers with names or social security numbers are visible. |
| 13. Acquisition, storage and disposal of clinical utensils and medicine/vaccines | More systematic control and documentation of refrigerator temperature, expiration dates on utensils, of cooling cabinet, and of lung function and pulse oximetry devices. |
| 14. Hygiene | The GP uses hand disinfectant more often.  More simple procedure for cleaning gynecological instruments (they previously did too much). |
| **Clinic 8** | |
| 2. Use of good clinical practice (Diabetes, COPD and a specific vulnerable group) | Made a new local procedure for dementia patients (their chosen vulnerable group) |
| 3. Adverse events | Adverse events have become a regular topic at monthly meetings. |
| 5. Prevention of confusion of patient’s identity | Personnel ask patients for social security number when performing tests. |
| 7. Paraclinical tests | Use of reminder functionality in the computer system to ensure monitoring of incoming test results and patients getting the result.  Keep a copy of the test requisition form for SMEAR examinations and check on a regular basis that patients with deviant test results have been informed.  Ensure that all test results (deviant or normal) are given to the patients (previously it was only deviant results). |
| 9. The patient health record, data safety and confidentiality | Lock cabinets in the patient waiting area. |
| 14. Hygiene | Renovated the room where instruments are cleaned with well-defined areas for ‘clean’ and ‘not clean’.  Purchased a new dishwasher for cleaning instruments.  Use bags for instruments after use of the dry sterilizer. |
| 15. Management and operational activities | Made an annual planning wheel displaying tasks in the clinic on a monthly basis. |
| **Clinic 10** | |
| 2. Use of good clinical practice (Diabetes, COPD and a specific vulnerable group) | More systematic procedures for COPD including annual check-ups. |
| 4. Patient evaluations | Changed telephone system to one with queue function (due to long waiting times and difficulties getting through). |
| 5. Prevention of confusion of patient’s identity | GP places test-samples and social security number together in small boxes and the nurse labels them (before they were placed on the table together, but with the risk of being mixed up).  The nurse asks for patient’s social security number when performing tests. |
| 7. Paraclinical tests | New procedure for ensuring follow-up on smear tests: Use trays for requisition, and does not remove them until the patient has received the result (negative or positive). |
| 8. Emergency response and cardiac arrest | Procured information on correct intervals for control and renewal of the defibrillator and made a schedule with fixed timespans for future controls. |
| 10. Availability | Changed telephone system to one with queue function (due to long waiting times and difficulties getting through). |
| 13. Acquisition, storage and disposal of clinical utensils and medicine/vaccines | More systematic control of expiration dates of medicine and vaccines and of refrigerator temperature.  Thorough clean-up of equipment (e.g. disposal of syringes past expiration date).  Regular control of medical bag using a newly created list of needed content. |
| 14. Hygiene | Added disinfection between washing of instruments and autoclavation.  Acquired special suits to use in case of contagious patients. |
| **Clinic 11** | |
| 5. Prevention of confusion of patient’s identity | Meticulously ask for patient’s social security number when performing tests. |
| 7. Paraclinical tests | New, clear procedures to ensure that deviant test result are delivered to the patient. |
| 13. Acquisition, storage and disposal of clinical utensils and medicine/vaccines | Purchased an electronic thermometer with data-logging for measuring refrigerator temperature (they found out that the temperature was unstable and intend to buy a new refrigerator). |
| 14. Hygiene | No change in routines, but thorough cleaning and clear-out of clinic before the survey visit. |
| **Clinic 12** | |
| 2. Use of good clinical practice (Diabetes, COPD and a specific vulnerable group) | Clarification of the task division between GPs and nurses in local procedures for diabetes and COPD.  Clearer task division regarding patients with psychiatric problems and alcohol and drug abuse (their chosen vulnerable group). |
| 5. Prevention of confusion of patient’s identity | More meticulously ask for patient’s name.  Always label a test sample container with the patient’s social security number before putting it aside. |
| 6. Prescription of medicine and renewal of prescriptions | New procedure for renewal of medicine: Patients can only renew some types of medicine prescriptions by having a consultation in the clinic. |
| 7. Paraclinical tests | Use of reminder functionality in the computer system to ensure monitoring of incoming test results and patients getting the result.  Stopped cultivating urine samples themselves, now sending the samples to the lab instead. |
| 8. Emergency response and cardiac arrest | Purchased a heart defibrillator.  Updated the emergency medical bag and ensure with a logbook that it is systematically controlled. |
| 9. The patient health record, data safety and confidentiality | Removal of papers with names or social security numbers from public sight.  Purchased a shredder and use it frequently. |
| 10. Accessibility | Engaged an extra part time secretary to ease patients’ phone access.  Opened a Facebook site with news and information. |
| 13. Acquisition, storage and disposal of clinical utensils and medicine/vaccines | Hazardous waste now taken to the pharmacy and no longer dropped at the waste disposal site for combustion.  More systematic control and documentation of expiration dates of medicine and vaccines. |
| 14. Hygiene | Changed to single-use equipment (to avoid time consuming sterilization and disinfection procedures and costly equipment). This also means that the small surgery they used to do can no longer take place in the clinic and is therefore referred to a specialist.  Blood pressure monitors lent to patients for measurement at home are now cleaned every time the apparatus is returned to the clinic.  More frequent cleaning of the tourniquet, the couch, and the electrocardiogram meter.  Toys removed from the waiting room. Now only paper and pencil at disposal, and the pencils are replaced every month.  Installation of a hand dryer instead of towels at the patients’ toilet.  Set up a room for handling contagious patients and a procedure for scheduling them at the end of the day. |
| 15. Management and operational activities | Use an annual planning wheel to structure the administration of the clinic. |
| 16. Hiring, introduction and competency development | Made an introduction program for new staff. |
